# Feasibility, acceptability and preliminary effects of a social network-based, peer-led HIV self-testing intervention among men in two Ugandan fishing communities, 2022

**DOI:** 10.1186/s13690-025-01511-9

**Published:** 2025-01-24

**Authors:** Joseph KB Matovu, Aisha Twahiri Namwama, Linda Kemigisha, Geoffrey Taasi, Jennipher Nakabugo, Julius Wandabwa, Laura M. Bogart, Nuraan Fakier, Rhoda K. Wanyenze, Peter Olupot-Olupot, Joshua Musinguzi, David Serwadda

**Affiliations:** 1 Faculty of Health Sciences, Busitema University, Mbale, Uganda; 2 Makerere University School of Public Health, Kampala, Uganda; 3 Ministry of Health, Kampala, Uganda; 4 RAND Corporation, Santa Monica, CA USA; 5 European & Developing Countries Clinical Trials Partnership, Cape Town, South Africa

**Keywords:** Peer-led, HIVST, Men, Fishing communities, Uganda

## Abstract

**Background:**

Social network-based interventions can improve uptake of health interventions. However, limited evidence exists on their feasibility and acceptability in fishing community settings. We assessed the feasibility, acceptability and preliminary effects of a social network-based, peer-led HIV self-testing (HIVST) intervention among men in Uganda.

**Methods:**

The *PE*er-led HIV*ST* intervention *for MEN* (PEST4MEN) is a pilot intervention conducted among men in Kalangala and Buvuma districts. Baseline data were collected in July 2022 and follow-up data in September 2022. The intervention was implemented through 22 trained lay men (“peer-leaders”) who received training in HIVST use and distribution processes and requested to refer at least 20 male members from their social networks for study eligibility screening. To be eligible, men had to be aged 15 years or older with unknown or HIV-negative status. After the baseline interview, men were requested to pick two oral fluid-based HIVST kits from their peer-leaders. The intervention was deemed feasible if peer-leaders gave-out > 80% of the kits and acceptable if > 80% of the kits’ recipients used them to self-test for HIV. At the follow-up interview, newly diagnosed HIV-positive men were asked if they had linked to HIV care. Data were descriptively analyzed using STATA version 16.0.

**Results:**

Of 475 screened men, 400 (84.2%) met the eligibility criteria and completed the baseline interview. Of these, 56.7% (*n* = 227) were engaged in fishing or fishing-related activities. At follow-up, 361 men (90.2%) were interviewed; 98.3% (*n* = 355) received at least one kit from their peer-leaders. Nearly all (99.1%, *n* = 352) kits’ recipients used them to self-test for HIV. Of the 352 HIV self-testers, 51 men (14.5%) had reactive (positive) HIV self-test results. Nearly one-third of the HIV self-tested men (31.4%, *n* = 16) were first-time HIV-positive testers. Of these, 87.5% (*n* = 14) went for confirmatory HIV testing, 50.0% (*n* = 7) were confirmed as HIV-positive and 71.4% (*n* = 5) were linked to HIV care.

**Conclusion:**

Our peer-led HIVST intervention was feasible and acceptable and identified newly diagnosed HIV-positive men who were linked to HIV care. However, while these results are promising, we recommend additional research in a randomized controlled trial prior to the eventual roll-out of this intervention.

**Trial registration:**

ClinicalTrials.Gov: NCT05685498 (retrospectively registered on January 17, 2023).

**Table:** 

Text box 1. Contributions to the literature
• Efforts to reach men with HIV testing services in the fishing communities have been hampered by men’s reluctance to utilize health facility-based services.
• Evidence suggests that community-based interventions that aim to reach men where they are can effectively improve uptake of HIV services among men.
• We used locally trained men to distribute HIV self-test kits to fellow men in existing social networks, with nearly universal uptake of HIVST services.
• Our intervention identified men with previously undiagnosed HIV infection who were eventually linked to HIV care. This suggests that the use of locally trained men to reach fellow men with HIVST services not only improves identification of newly diagnosed HIV-positive men but also presents a great opportunity to link such men to HIV care.
• If implemented at scale, this approach has the potential to improve HIV testing uptake and linkage to HIV care among men in remote fishing communities, who would have been missed through conventional, health facility-based services.

## Background

In the Eastern and Southern Africa region, the region with the highest HIV prevalence in sub-Saharan Africa, reaching men with HIV testing services remains largely elusive [1]. As shown in previous studies, men and boys aged 15 years or older living with HIV are 20% less likely than women and girls living with HIV to know their HIV status, and 27% less likely to be accessing treatment [1, 2]. Those who know their HIV-positive status and are enrolled in antiretroviral therapy programs are 70% more likely to die than women because of their poor adherence to treatment [1]. Several studies have attributed these gender differences to male masculinity norms [3–5], lack of time to go for HIV testing services [6], and men’s reluctance to utilize facility-based HIV testing services [7]. Also, men tend to have limited HIV testing opportunities than women who can access HIV testing services through antenatal care or prevention of mother-to-child transmission of HIV services [8, 9]. Collectively, these findings call for a need to implement innovative HIV testing and linkage to HIV care strategies that can reach men in settings where they work or live [7, 10].

HIV self-testing (HIVST)– a process in which a person collects his or her own specimen (either oral fluid, sometimes called saliva, or else blood from a finger prick), then performs a rapid HIV test and interprets the result [11]– is a promising strategy to increase HIV testing uptake and linkage to HIV care among populations that are usually missed through conventional, health facility-based HIV services [12, 13]. Several strategies– including door-to-door delivery of kits by trained lay HIV self-test kits distributors, use of peer-educators and secondary distribution of kits via sexual partners and female sex workers– among others, have been used to reach diverse population groups, usually outside of formal health facilities [12, 14, 15]. One promising approach that has got the potential to reach men in hard-to-reach communities with HIV testing services is the use of social network-based approaches [16–20]. Social networks can be defined as groups of people who are closely connected to each other and who usually consult each other on several aspects, including socio-economic and health matters. Because of their close social ties, health information can be diffused effectively and quickly through these networks. Evidence from previous studies show that peer-led, social network-based approaches can enhance HIVST uptake and linkage to HIV care services [21, 22, 24–27]. However, until recently [21, 22], this level of evidence has only been established for men who have sex with men [18–20, 24], young people [25, 27] and female sex workers [26] but not among heterosexual men living in fishing communities in sub-Saharan Africa.

Evidence from studies conducted in Uganda [21–25] and elsewhere [26–28] suggests that peer-led HIVST is acceptable and can improve HIV testing and linkage to HIV care services among diverse populations. However, most of these studies were not conducted in fishing communities [23–28], making their findings to be less generalizable to fishing community settings. Thus, further research is warranted to understand how to reach men with HIVST services in fishing communities that are usually located far away from the main health services. In this pilot interventional study, we assessed the feasibility, acceptability and preliminary effects of a peer-led HIVST intervention in two fishing communities located in two island districts within Lake Victoria to inform the design of a larger trial to assess population-level effects of peer-led HIVST in Ugandan fishing communities.

## Methods

### Study design and sites

The *PE*er-led HIV *S*elf-*T*esting intervention *for MEN* (PEST4MEN) is before-after, single-arm, pilot interventional study implemented in two fishing communities in two island districts of Kalangala and Buvuma, located within Lake Victoria in Uganda. Kalangala district is made up of 84 islands with an estimated population of 67,000 people. The district has 15 health facilities located on seven of the 84 islands, with the highest-level health facility (Kalangala Health Center IV) located on Buggala island, the biggest island in the district. Buvuma district is made up of 52 islands with an estimated population of 20,000 people. Only four islands have a health center, including Buvuma, the biggest island, which hosts the highest-level health facility (Buvuma Health Center IV) in the district. The study was conducted at Mwena fishing community on Buggala island, Kalangala district, and Kasaali ‘B’ fishing community on Buvuma island, Buvuma district. The two districts were selected because of their island location and also because of their higher-than-average national HIV prevalence. Although accurate HIV prevalence estimates among the fisherfolk in the two districts are not available, some reports cite an adult (15–49 years) HIV prevalence level of 14% in Buvuma [29] and 18.8% in Kalangala district [30], both much higher than the national average of 5.5% among adults aged 15–49 years [31]. It is important to note that although the Ugandan Ministry of Health has officially rolled out the provision of HIVST services in all government health facilities; at the moment, the coverage of HIVST services delivered through community-based distribution channels remains unknown.

### Intervention description

The design of the PEST4MEN intervention was informed by the Information, Motivation and Behavioral Skills (IMB) model [32]. The IBM model posits that if individuals are well informed, motivated to act, and possess the requisite behavioral skills for effective action, they will be likely to take on the recommended action response [32]. Using this model, we assumed that if male social network members received information about HIVST from trained peer-leaders, who were members of their own social networks, they would be motivated to self-test for HIV. Evidence shows that people tend to trust information that they receive from close friends or trusted peers within their own social networks [33]. We also assumed that peer-leaders would be willing to distribute HIVST kits to their male social network members, based on our previous research on this subject [34] and that they would be willing to train their social network members in how to use the kits as well as in how to read and interpret HIV self-test results, thereby enhancing their HIVST behavioral skills. The following sub-sections describe the different steps that were followed during the design and implementation of the PEST4MEN intervention.

### Formative research and peer-leaders’ selection

The PEST4MEN intervention uses existing social networks to reach men with HIVST services. Eighteen male-only social groups/networks were identified through a formative study that was conducted prior to the implementation of the intervention. The formative study was conducted using focus group discussions (FGDs) to document existing social networks of men in each fishing community, identify community perceptions about the peer-led HIVST intervention, and document preferred qualities of male HIV self-test kits distributors. Community meetings were then held to select 1–2 potential male HIV self-test kits distributors (hereafter referred to as “peer-leaders”) from each social network, following a set of pre-defined qualities that were identified through the formative study. Specifically, to be selected as a peer-leader, men had to: (a) be aged 18 years or older, resident in the targeted fishing community; (b) be able to read and write in English and *Luganda*, the predominant local language; (c) have a high level of trust as judged by community residents; (d) be known to keep secrets; (e) be popular within their networks, and (f) be considered to be approachable by community residents. The community meetings were convened by a member of the study team. At each social network group meeting, we asked men to nominate, among themselves, three names of other men who had the above-mentioned qualities, followed by voting through show of hands. The candidate with the highest number of votes was selected as the peer-leader for that particular group. The same procedures were followed in selecting peer-leaders across all the 18 social networks. Some social networks that were too big to be “led” by one peer-leader (e.g. those with 50 + members) had the option of selecting a second peer-leader. We did not assign any weights to the peer-leader qualities during the peer-leader selection process; meeting participants agreed through mutual consensus if the nominated male member of their social network met these qualities.

### Peer-leaders’ training and selection of social network members

Selected peer-leaders received three days’ training in HIVST procedures (e.g., how to check the expiry date on the HIVST package, how to open the HIVST package, how to remove the HIV self-test kit from the HIVST package, how to open the bottle with the HIV testing solution, how to place the bottle within its stand at the time of self-testing, how to obtain the oral swab, and how to time the 20 min needed to perform the test) with an in-person demonstration of these procedures by a member of the study team. During the training, peer-leaders were introduced to basic counseling skills, how to complete the referral forms, the importance of confirmatory HIV testing among HIV-positive self-testers, peer-to-peer counseling, and how to complete the study tracking forms. The training took three days and included role-plays and demonstration of the above-mentioned procedures by a peer-leader standing in front of other trainees. The other trainees helped to point out errors made by the peer-leader (if any) and these were discussed among all trainees, including how to avoid them. At the end of the training, each peer-leader was asked to refer to the study team at least 20 social network members that they personally knew and who they interacted with at least once a week. The emphasis on weekly interaction between the peer-leaders and their social network members was because the distribution of HIV self-test kits required that peer-leaders physically meet their social network members at agreed-upon venues to distribute the kits. It is through these physical meetings that the peer-leaders would educate their social network members about the required HIVST procedures and demonstrate to them how the HIVST exercise is conducted. A physical meeting also gave the social network members the opportunity to ask questions where they were not clear about the processes or on how to read or interpret their HIV self-test results, or on what to do upon receiving a HIV-positive self-test result. All referred social network members were screened for study eligibility and those who were found to be eligible were administered a baseline interview (see ‘*study population*’ below).

### Baseline interview and distribution of HIV self-test kits

**Fig. 1:**
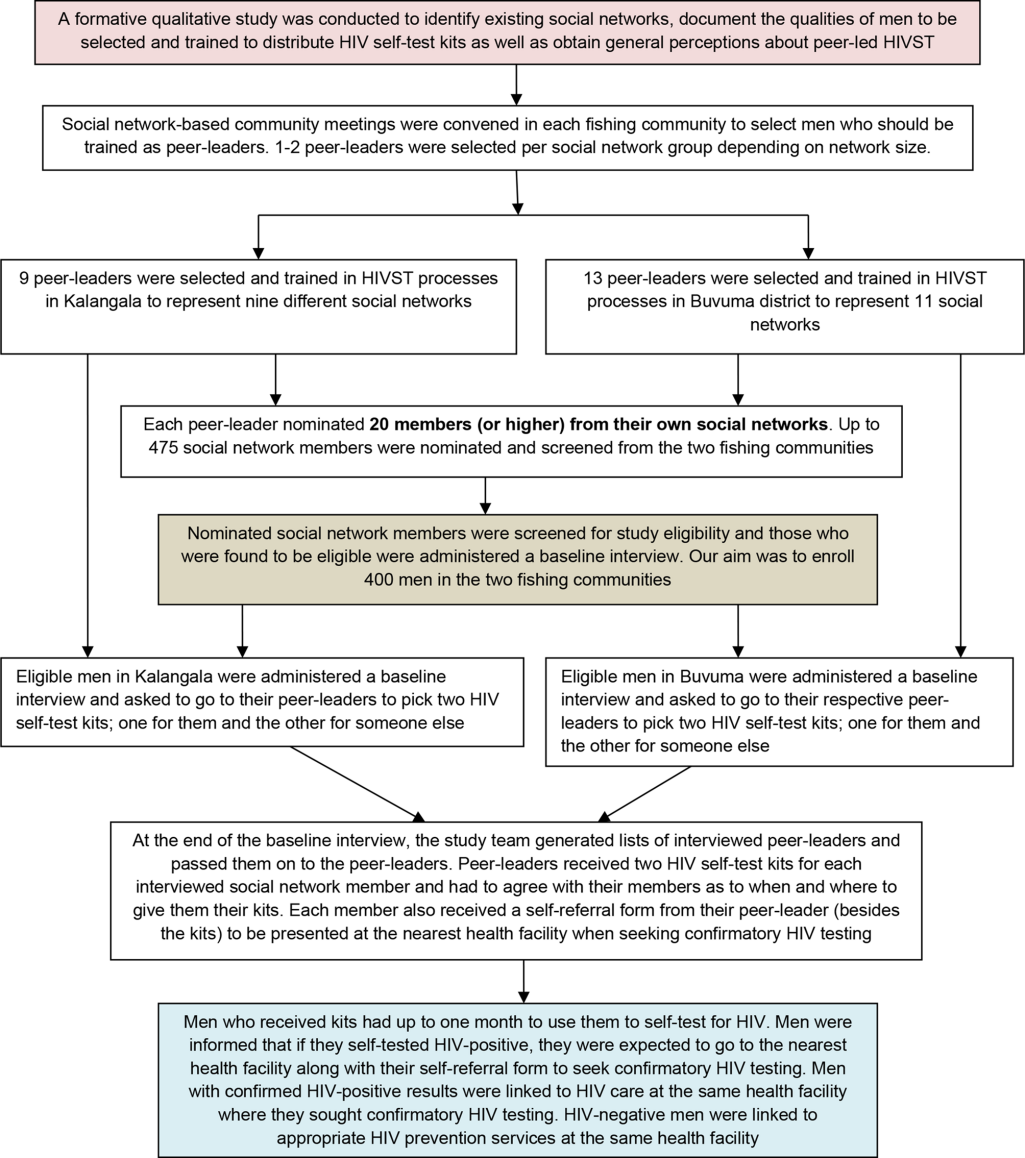
Peer-led HIVST study-related implementation processes

Eligible social network members (see ‘*study population*’ below) were administered a baseline questionnaire and later sent to their respective peer-leaders to pick their HIV self-test kits. The kits were distributed through the peer-leaders rather than by members of the study team because the intervention was designed to be peer-led. All eligible participants had to go to the peer-leader who nominated them to obtain their HIV self-test kits. To facilitate this process, each peer-leader received the list of social network members who had been enrolled into the study out of those that they had referred for study eligibility screening. For each enrolled member, a peer-leader was given two kits, one for the member and the other for someone else in the member’s social network. We left it to the discretion of the peer-leader and their social network members to decide when and where to meet to receive the kits, as long as this was done within a period of one month from the baseline interview. Peer-leaders received approximately $5.5 as time compensation to facilitate the distribution of HIV self-test kits to social network members and completion of the necessary paper-work to document the characteristics of the recipients. See Fig. 1 for a detailed description of the HIV self-test kits distribution process.

### HIVST and linkage to HIV care

The study team worked with a Liaison Nurse Counselor at a participating government health facility in each district. The role of this Nurse Counselor was to provide additional information to the HIV self-testers (before, during or after HIVST), to arrange for confirmatory HIV testing among men who received a HIV-positive self-test result, and to link confirmed HIV-positive men to HIV care as appropriate. All social network members were given the telephone contact of the Liaison Nurse and were advised to contact her for any additional information beyond that which they received from their peer-leaders. Men who received HIV self-test kits could opt to conduct the HIVST exercise alone or seek assistance from their peer-leaders. All respondents received a self-referral form through their peer-leaders which they were asked to present to the Liaison Nurse Counselor if they went to the health facility for confirmatory HIV testing. Individuals with confirmed HIV-positive results were linked to HIV care with the support of the Liaison Nurse, following the Uganda Ministry of Health’s consolidated HIV prevention and treatment guidelines [35]. All the social network members who received HIV self-test kits were requested to use them within a period of one month and to return used kits to the study team at the next study visit for re-reading by a member of the study team.

### Study population

This study was conducted among eligible men who were referred to the study team from existing social networks by trained peer-leaders. A social network was defined as any loose grouping of people (in this case, men) who lived or worked together and met daily or occasionally for social or economic purposes. This study focused on male-only social networks and enrolled men in general, regardless of whether or not they engaged in fishing or fishing-related activities. To be enrolled into the study, men had to be nominated by a trained peer-leader from an existing social network, aged 15 years or older, be HIV-negative at last test or be of unknown HIV status. Ever-tested men had to have last tested for HIV four or more (4+) months ago, prior to enrolment. It is important to note that peer-leaders were not interviewed as part of this intervention; only their social network members.

### Sample size determination

We used quota sampling techniques to select men from each social network, based on experiences from a prior feasibility study [21]. To select men, each peer-leader was asked to refer at least 20 members from their social networks for study eligibility screening. There were 22 trained peer-leaders in total; nine in Kalangala and 13 in Buvuma district. The nine peer-leaders in Buvuma referred 215 men to the study team while the 13 peer-leaders in Buvuma referred 260 men for a total of 475 men in the two study districts. When these men were.

screened for study eligibility, 400 men met the study eligibility criteria and were administered a baseline interview.

### Data collection procedures and methods

Baseline data collection for the PEST4MEN study took place in July 2022 with follow-up data collected two months post-baseline in September 2022. Data were collected using a structured, pilot-tested questionnaire configured in *KoboCollect* application and uploaded on mobile phones. Data were collected through face-to-face interviews by trained data collectors with experience in the conduct of quantitative surveys. At the baseline visit in July 2022, data were collected on respondents’ socio-demographic and behavioral characteristics (e.g. number of sexual partners in the past 3–6 months; prior HIV testing experiences, condom use at last sex, alcohol use before sex, etc.) as well as preferences about HIVST. At the follow-up interview in September 2022, data were collected on whether or not men received HIV self-test kits from their peer-leaders, where they received the kits from, and what relationship they had with the person who gave them the kits (to confirm that they actually received the kits from their peer-leaders). Individuals who received HIV self-test kits were asked whether or not they used them to self-test for HIV. Individuals who reported a HIV-positive self-test result were asked if it was their first time to test HIV-positive and if so, whether or not they sought confirmatory HIV testing. All those who reported that they sought confirmatory HIV testing were asked if they received their confirmed HIV test results. If they were confirmed as being HIV-positive, participants were asked if they were linked to HIV care, including how soon after confirmatory HIV testing. Male social network members provided written informed consent prior to the baseline interview and were provided with a time compensation of UGX 20,000 (~ US$5.5, based on the December 2024 exchange rates) at the end of each interview. Each interview lasted approximately 60 min.

### Measures

Three primary outcomes were assessed: feasibility of the intervention, acceptability of the intervention, and the preliminary effects of the intervention on HIV testing uptake and linkage to HIV care. Borrowing from our previous study in which similar measures were applied [21], the PEST4MEN was deemed feasible if > 80% of the kits given to the peer-leaders were distributed to men within their social networks and acceptable if > 80% of the men (social network members) who picked kits from their peer-leaders actually used them to self-test for HIV. To measure the preliminary intervention effects, all men who received HIV-positive self-test results were asked if they were testing HIV-positive for the first time. First-time HIV-positive self-testers were asked if they went for confirmatory HIV testing, and of these, what proportion linked to HIV care after confirmation of their HIV-positive status.

### Data analysis

We conducted descriptive analyses to determine the proportion of individuals who used the kits to test for HIV among those that received the kits from their peer-leaders. We measured the proportion of newly diagnosed HIV-positive individuals who sought confirmatory HIV testing services at the nearest health facilities and, among those confirmed as HIV-positive, what proportion was linked to HIV care within two months of their confirmed HIV-positive status. Comparisons between proportions were made using Pearson’s Chi-square tests. Data were analyzed using STATA statistical software, version 16.0.

### Ethical approval

Ethics approval for the PEST4MEN study was sought from Makerere University School of Public Health Research and Ethics Committee (Protocol #SPH-2021-158). The approved protocol was cleared by the Uganda National Council for Science & Technology as per national research guidelines (#HS2034ES). No informed consent form was administered at the time of screening; only after men had been considered to be eligible for study enrolment, just before the survey interview. Although we intended to enroll men aged 15 + years, we did not identify any eligible men aged 15–17 years. Thus, we enrolled men aged 18 + years and these men provided written informed consent as per national research guidelines.

## Results

**Fig. 2:**
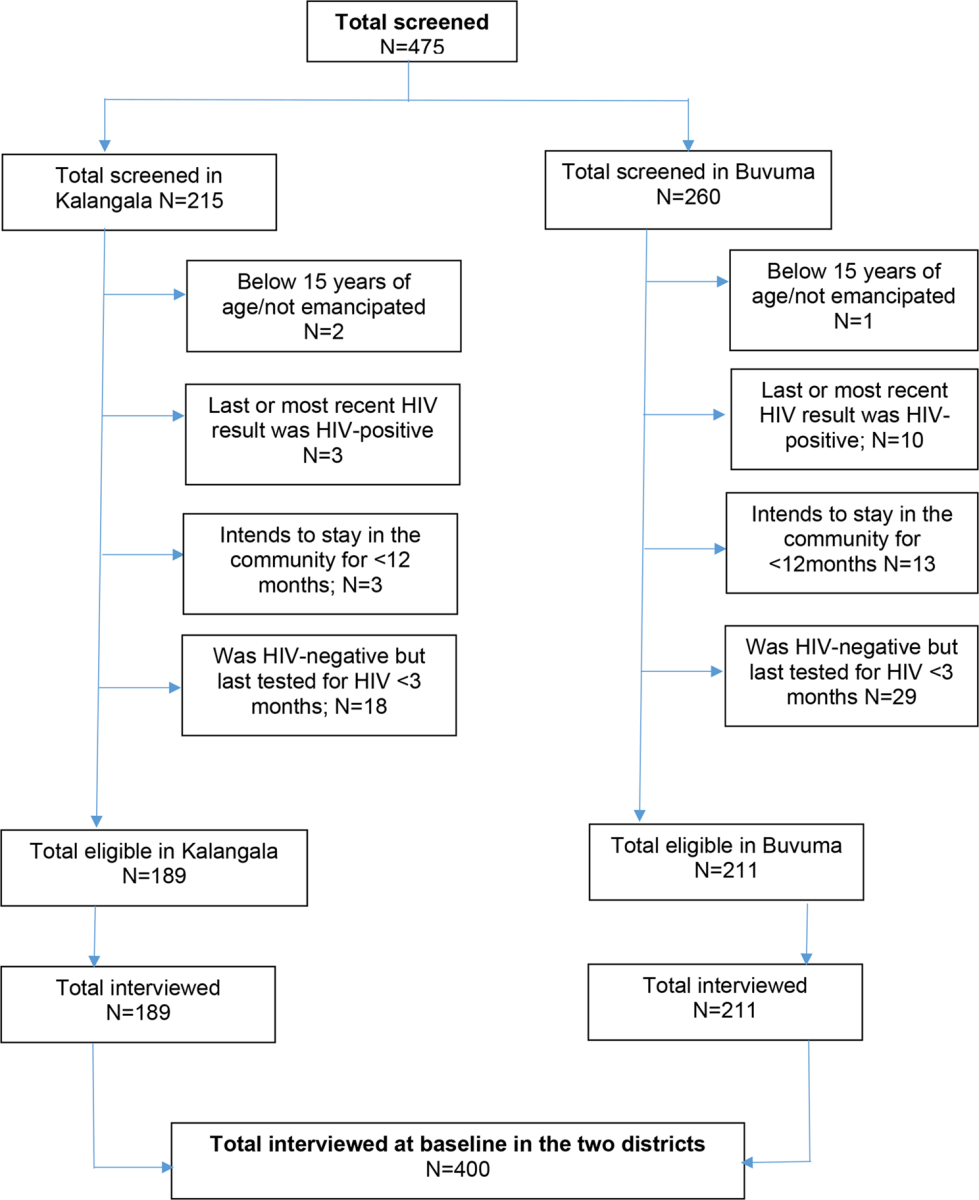
Study flowchart

Of 475 male social network members screened for study eligibility, 215 were from Kalangala while 260 were from Buvuma. Of these, 12.1% (*n* = 26) in Kalangala and 18.8% (*n* = 49) in Buvuma district (or 15.8%, *n* = 75, in total)– were ineligible for study enrolment. The reasons for being ineligible included having last tested for HIV within the past three months (62.7%, *n* = 47), being HIV-positive (17.3%, *n* = 13), or other reasons (20.0%, *n* = 15). After eliminating those who were not eligible for study enrolment, 189 of the social network members in Kalangala and 211 of those in Buvuma were enrolled into the study for a total of 400 respondents (Fig. 2).

### Baseline characteristics

Table 1 shows the characteristics of the 400 male social network members that were enrolled into the study at baseline. A greater majority of the men in both districts (67.9%, *n* = 272) were aged between 18 and 34 years; nearly two-thirds (64.2%, *n* = 257) had primary education, while more than half (58.2%, *n* = 233) were currently married. Nearly 41.0% (*n* = 163) of social network members could not read text written in *Luganda*, the main local language spoken in their community; with a higher proportion of men in Buvuma being unable to read text written in the local language than men in Kalangala district (Buvuma: 52.1%, *n* = 110 vs. Kalangala: 28.0%, *n* = 53; *P* < 0.0001). Slightly over half of the male social network members (56.7%, *n* = 227) were engaged in fishing or fishing-related activities. A majority of the respondents (82.0%, *n* = 328) had ever tested for HIV.

**Table 1:** Baseline socio-demographic characteristics of men in Kalangala and Buvuma, overall and by study district (July 2022)

Characteristic	Kalangala (*N* = 189, %)	Buvuma (*N* = 211, %)	Total (*N* = 400, %)
**Age-group**			
18–24 years	59 (31.2)	66 (31.3)	125 (31.2)
25–34 years	68 (36.0)	79 (37.4)	147 (36.7)
35–44 years	44 (23.3)	50 (23.7)	94 (23.5)
45 + years	18 (9.5)	16 (7.6)	34 (8.5)
**Highest level of education attained**			
No education	10 (5.3)	19 (9.0)	29 (7.2)
Primary education	125 (66.1)	132 (62.6)	257 (64.2)
Post-primary	54 (28.6)	60 (28.4)	114 (28.5)
**Marital status**			
Never married/not in any relationship	13 (6.9)	13 (6.2)	26 (6.5)
Never married but in relationship	44 (23.3)	37 (17.5)	81 (20.2)
Ever married, not in a relationship	28 (14.8)	11 (5.2)	39 (9.7)
Ever married, in a relationship	17 (9.0)	4 (1.9)	21 (5.2)
Currently married	87 (46.0)	146 (69.2)	233 (58.2)
**Ability to read text in the local language** (*Luganda*)			
No, cannot read text in the local language at all	53 (28.0)	110 (52.1)	163 (40.7)
Yes, but reads text in the local language with difficulty	53 (28.0)	48 (22.7)	101 (25.2)
Yes, reads text in the local language fluently	83 (43.9)	53 (25.1)	136 (34.0)
**Occupation**			
Fishing	65 (34.4)	88 (41.7)	153 (38.2)
Fishing-related activity	33 (17.5)	41 (19.4)	74 (18.5)
Business/commercial	39 (20.6)	34 (16.1)	73 (18.2)
Other occupation	52 (27.5)	48 (22.7)	100 (25.0)
**Ever tested for HIV**			
Yes	168 (88.9)	160 (75.8)	328 (82.2)
No	21 (11.1)	51 (24.2)	71 (17.8)
**Mobile phone ownership**			
Yes	168 (88.9)	183 (86.7)	351 (87.7)
No	21 (11.1)	28 (13.3)	49 (12.2)

### Feasibility of the intervention

**Fig. 3:**
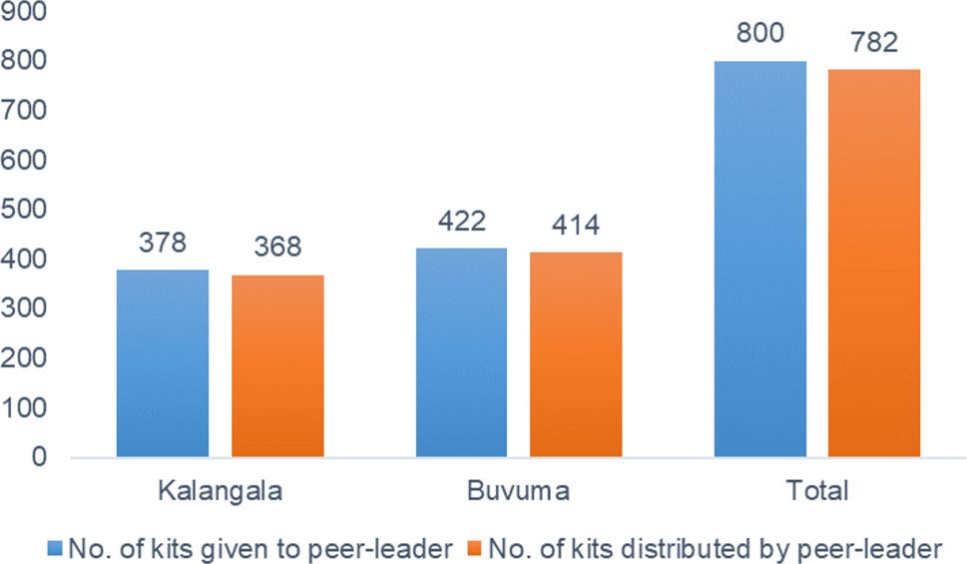
Number of kits given to peer-leaders by the study team and number of kits that they distributed to their social network members in Kalangala and Buvuma (September 2022)

Figure 3 shows the number of HIV self-test kits given to the peer-leaders by the study team and the number of kits that they gave out to their social network members. Overall, peer-leaders received 800 kits to distribute to their social network members. Within two months, peer-leaders distributed 97.7% (*n* = 782) of the kits that were given to them by the study team; more in Buvuma (98.1%, *n* = 414) than in Kalangala district (97.3%, *n* = 368).

At the subsequent follow-up visit in September 2022, we interviewed 90.2% (*n* = 361) of the 400 social network members enrolled at baseline. Of those interviewed at follow-up, nearly all men (98.3%, *n* = 355) reported that they received at least one kit from their peer-leaders. Of these, 80.3% (*n* = 285) received two kits while 19.7% (*n* = 70) received one kit. When asked where they received the kits from, 49.6% (*n* = 176) of all recipients reported that they received them from their peer-leaders’ homes, 26.8% (*n* = 95) had the kits delivered to their homes by a peer-leader, 16.6% (*n* = 59) had the kits delivered to their own work places by a peer-leader, while 4.2% (*n* = 15) picked the kits from the peer-leaders’ work places. Male social network members from Buvuma were almost three times as likely to report that they picked the kits from their peer-leaders’ homes than those in Kalangala (odds ratio [OR] = 2.79; 95% confidence interval [95%CI]: 1.77, 4.39). On the contrary, men in Kalangala had twice the odds of having the kits delivered to their homes by the peer-leaders than those in Buvuma (OR = 2.07; 95%CI: 1.24, 3.45). After receiving the kits, 74.6% (*n* = 265) reported that they were contacted by their peer-leaders to check if they had used them to self-test for HIV while 51.5% (*n* = 183) reported that they were provided with information on the existing referral networks. When asked about how comfortable they were to receive HIV self-test kits from their peer-leaders, 87.9% (*n* = 312) reported that they were comfortable or very comfortable to do so. When asked if they would recommend that trained peer-leaders continue to distribute HIV self-test kits in the fishing communities, 98.0% (*n* = 348) responded in the affirmative with a similar proportion of men in Buvuma (97.8%, *n* = 177) and Kalangala (98.3%, *n* = 171) responding in this way (Table 2).

**Table 2:** Characterizing the HIV self-test kits distribution processes among social network members by peer-leaders in Kalangala and Buvuma districts (September 2022)

Variables	Kalangala (*N*, %)	Buvuma (*N*, %)	Total (*N*, %)
**No. of men interviewed at baseline**	189	211	400
**Number (%) of men interviewed at follow-up**	175 (92.6)	186 (88.1)	361 (90.2)
**Number (%) of men interviewed at follow-up who received at least one kit from their peer-leaders**	**174 (99.4)**	**181 (97.3)**	**355 (98.3)**
	*N* = 174	*N* = 181	*N* = 355
**Number of kits that men received from their peer-leaders**			
1 kit	25 (14.4)	45 (24.9)	70 (19.7)
2 kits	149 (85.6)	136 (75.1)	285 (80.3)
**Place where you received the HIV self-test kits from the peer-leader**			
Peer-leader’s home	64 (36.8)	112 (61.9)	176 (49.6)
At own home	59 (33.9)	36 (19.9)	95 (26.8)
At own work place	38 (21.8)	21 (11.6)	59 (16.6)
Peer-leader’s work place	6 (3.4)	9 (5.0)	15 (4.2)
At church	0 (0.0)	1 (0.5)	1 (0.3)
Other	7 (4.0)	2 (1.1)	9 (2.5)
**After you received the kits**,** did the peer-leader call you/follow-up with you to check if you had used the kit to self-test for HIV?**			
Yes	124 (71.3)	141 (77.9)	265 (74.6)
No	50 (28.7)	40 (22.1)	90 (25.3)
**After you received the kits**,** did the peer-leader provide you with information on existing referral networks just in case you tested HIV-positive and needed to link to HIV care?**			
Yes	82 (47.1)	101 (55.8)	183 (51.5)
No	92 (52.9)	80 (44.2)	172 (48.4)
**How comfortable was it for you to receive HIV self-test kits from this person?**			
Comfortable	104 (59.8)	71 (39.2)	175 (49.3)
Very comfortable	49 (28.2)	88 (48.6)	137 (38.6)
Uncomfortable	10 (5.7)	7 (3.9)	17 (4.8)
Very uncomfortable	11 (6.3)	13 (7.8)	24 (6.8)
Not sure	0 (0.0)	2 (1.1)	2 (0.6)
**Would you recommend that HIV self-test kits continue to be distributed in the fishing communities by trained peer-leaders?**			
Yes	171 (98.3)	177 (97.8)	348 (98.0)
No	3 (1.7)	4 (2.2)	7 (2.0)

### Acceptability of the intervention

Table 3 shows HIV self-testing uptake among men who received HIV self-test kits from their peer-leaders. Of the 355 men who reported that they received HIV self-test kits from their peer-leaders, 99.1% (*n* = 352) reported that they used them to self-test for HIV. Of these, 45.2% (*n* = 159) used the kits on the same day of receiving them; 21.3% (*n* = 75) used the kits on the next day, while 14.8% (*n* = 52) used them within 2–4 days of receiving them. Of those that self-tested for HIV, 29.5% (*n* = 104) reported that they self-tested under the direct supervision of someone else; however, 96.6% (*n* = 340) of all self-testers performed the test on their own.

**Table 3:** Characterizing the HIV self-testing processes among the men who received and used HIV self-test kits from their peer-leaders in Kalangala and Buvuma districts (September 2022)

Variables	Kalangala (*N*, %)	Buvuma (*N*, %)	Total (*N*, %)
**No. of men that received kits**	174	181	355
**Number (%) of men that used the kits to self-test for HIV**	174 (100)	178 (98.3)	352 (99.1)
	*N* = 174	*N* = 178	*N* = 352
**How long after you received the kit did you use it to self-test for HIV?**			
Used it immediately/same day	68 (39.1)	91 (51.1)	159 (45.2)
Used it next day	39 (22.4)	36 (20.2)	75 (21.3)
Used it 2–4 days later	22 (12.6)	30 (16.8)	52 (14.8)
Used it 5–7 days later	11 (6.3)	10 (5.6)	21 (6.0)
Used it > 1 week after receiving it	34 (19.5)	11 (6.2)	45 (12.8)
**Did you perform the HIVST exercise under the direct supervision of anyone?**			
Yes	55 (31.6)	49 (27.5)	104 (29.5)
No	119 (68.4)	129 (72.5)	248 (70.4)
**Who actually conducted the oral HIV self-test?**			
Self	168 (96.5)	172 (96.6)	340 (96.6)
Spouse	2 (1.1)	0 (0.0)	2 (0.6)
Other person	4 (2.3)	6 (3.4)	10 (2.8)
**What was the HIV self-test result as read by a member of the study team?**			
HIV-positive	33 (20%)	15 (8%)	48 (14%)
HIV-negative	134 (77%)	151 (85%)	285 (81%)
Indeterminate	5 (3%)	1 (1%)	6 (2%)
Kit not yet returned	2 (1%)	11 (6%)	13 (4%)
**What was the HIV self-test result as read by the respondent?**			
HIV-positive	19 (11%)	14 (8%)	33 (9%)
HIV-negative	143 (82%)	150 (84%)	293 (83%)
Indeterminate	2 (1%)	1 (1%)	3 (1%)
Don’t know/Don’t remember	10 (6%)	13 (7%)	23 (7%)

Table 4 shows the level of agreement between two independent groups of HIV self-test results readers (i.e., respondents or members of the study team). Both groups identified 8.5% (30/352) of the men as being HIV-positive and 77.3% (272/352) as being HIV-negative, giving an *observed level of agreement* of 0.878 (or 87.8%). However, there were variations in the total number of men identified as HIV-positive or HIV-negative by each group: respondents identified a total of 33 men as HIV-positive while study team members identified a total of 48 men as HIV-positive. On the other hand, respondents identified 293 men as HIV-negative while study team members identified 285 men as HIV-negative. Based on these variations, we estimated the *expected level of agreement* by chance to be 0.686 (or 68.6%). Using the formula recommended by McHugh for calculating the Kappa statistic [36], we obtained a Cohen’s Kappa coefficient (*κ*) of 0.611 (or 61.1%).

**Table 4:** Inter-rater agreement between the respondents’ self-reading of HIV self-test results and their reading by study team members in Kalangala and Buvuma districts (September 2022)

Reading by the respondent	Reading of the kit by a member of the study team				
	*HIV-positive*	*HIV-negative*	*Indeterminate*	*Kit not returned*	*Total*
*HIV-positive*	**30**	0	0	3	**33**
*HIV-negative*	9	**272**	3	9	**293**
*Indeterminate*	0	0	3	0	3
*Don’t know/don’t remember*	9	13	0	1	23
**Total**	**48**	**285**	6	13	352

### Preliminary effects of the intervention

Table 5 shows the number of HIV positive men by whether or not they were first-time or repeat HIV-positive testers and if they were first-time HIV-positive testers, whether or not they linked to HIV care. Overall, 14.5% (*n* = 51) of the self-tested men had reactive HIV self-test results. Of these, 31.4% (*n* = 16) were newly diagnosed while 68.6% (*n* = 35) were repeat HIV-positive testers who were already aware of their HIV-positive status (some of these men were already receiving HIV care) prior to study enrolment. Of the 16 newly diagnosed HIV-positive self-testers, 87.5% (*n* = 14) went for confirmatory HIV testing, 50.0% (*n* = 7) were confirmed as HIV-positive and, of those with confirmed HIV test results, 71.4% (*n* = 5) were initiated on ART.

**Table 5:** Distribution of HIV-positive men in Kalangala and Buvuma districts by whether or not they were newly diagnosed and, if newly diagnosed, whether or not they were linked to HIV care (September 2022)

Variable	Kalangala (*N* = 34)	Buvuma (*N* = 17)	Total (*N* = 51)
No. (%) of men who were already HIV-positive at the time of enrolment	25 (73.5)	10 (58.8)	35 (68.6)
No. (%) of men with a first-time reactive HIV self-test result	9 (26.5)	7 (41.2)	16 (31.4)
Did you go for confirmatory HIV testing? (Yes)	7 (77.8)	7 (100)	14 (87.5)
No. (%) of men with confirmed HIV-negative results	5 (71.4)	2 (28.6)	7 (50.0)
No. (%) of men with confirmed HIV-positive results	2 (28.6)	5 (71.4)	7 (50.0)
No. (%) of men with confirmed HIV-positive results who were linked to HIV care	1 (50.0)	4 (80.0	5 (71.4)

## Discussion

Our findings show that a social network-based, peer-led HIVST intervention is feasible and acceptable and identifies previously undiagnosed HIV-positive men who would not have known their HIV sero-positive status without this intervention [37–39]. Specifically, peer-leaders distributed 98% of the kits to their social network members; 99% of those who received the kits used them to self-test for HIV, while 31.4% of men living with HIV were first-time HIV-positive testers. Of first-time HIV-positive testers with confirmed results, nearly three-quarters were linked to HIV care. Collectively, these findings suggest that a peer-led HIVST intervention may be the game-changer needed to improve HIV testing and linkage to HIV care among men in remote fishing communities in Uganda.

Our finding that the peer-led HIVST intervention was feasible and acceptable is consistent with previous findings in similar populations [21–23] and lend credence to the need for community-based HIVST interventions, including those that train locally available men as community HIV self-test distributors [40–43]. The use of trained locally available men to distribute HIV self-test kits can help to improve men’s confidence in the use of HIV self-test kits and, as previously documented [21–23], men in the fishing communities can easily pick kits from their local distributors at any time of the day, rather than travel to far-off health facilities to pick them. Our findings show that nearly all men were willing to receive HIV self-test kits from trained male distributors in the community, and, indeed, nearly 88% reported that they were comfortable with receiving HIV self-test kits from them. Collectively, these findings suggest that the use of trained male distributors who are residents of the same community as the potential male recipients can help to improve acceptability and eventual use of the HIV self-test kits to self-test for HIV among men.

We found that nearly one-third of HIV-positive men were first-time HIV-positive testers, most of whom went for confirmatory HIV testing. Of these, half were confirmed as HIV-positive and nearly three-quarters were linked to HIV care. These findings suggest that our peer-led HIVST intervention can help to identify newly diagnosed HIV-positive men who can link to HIV care. However, it is important to note that half of the men who went for confirmatory HIV testing turned out to be HIV-negative. This prompted us to conduct a qualitative inquiry as to why this was the case. Our investigation revealed inconsistencies in the reading of HIV self-test results for five men. While the respondents read their results as HIV-negative, study team members read their results as HIV-positive after seeing a second weak band on the used kit. When these men went for confirmatory HIV testing, their results were confirmed as HIV-negative, which was consistent with the results that they (respondents) had read on the kit. This scenario is consistent with findings from previous studies in which stored kits were found to have developed a second weak band [44, 45] but when the concerned respondents were re-tested, all of them turned out to be HIV-negative. It is thus likely that re-reading of HIV self-test kits after initial use may not be justifiable as the results on the kit become unstable with time. Nevertheless, our finding that nearly three-quarters of first-time HIV-positive men with a confirmed HIV-positive status were linked to HIV care is a clear indication that if men can be motivated to seek confirmatory HIV testing, we will be able to link more HIV-positive men to HIV care.

We noted stark differences with regard to where and when men self-tested for HIV, with men in Buvuma preferring to self-test immediately while those in Kalangala delayed to self-tested for HIV. We don’t know exactly why men in these two districts behaved differently even if we used the same approach of distributing HIV self-test kits to them. However, if we reflect on our implementation experiences, we observed that men in Buvuma were more receptive to the intervention than those in Kalangala and this could explain the observed differences in HIVST behaviors but this observation is not exhaustive. Further research is therefore warranted to understand the differentials in HIV testing behaviors among men in both districts. Understanding these differentials is crucial for sustained HIV prevention efforts given that these delays could be a manifestation of other underlying HIV risk-behaviors that make men fear to test for HIV.

Lastly, the finding that the level of agreement between the respondents and members of the study team with regard to the reading and interpretation of HIV self-test results was 61% raises a serious public health concern. Based on McHugh’s [36] suggested interpretation of this level of agreement, we can estimate that there was a moderate level of agreement between the two groups of readers. These findings suggest a need for further training of potential users and, by implication, the peer-leaders, regarding how to read and interpret HIV self-test results. There is also a need to ensure that members of the research team receive adequate levels of training regarding how to read and interpret results to minimize cases of disagreement between study participants and members of the research team when it comes to the reading and interpretation of HIV self-test results.

This study had a number of limitations and strengths. The main limitation was the fact that this was a single-arm, before-after intervention without a comparison group. For this reason and, as noted elsewhere [46], our study outcomes may not be reliably attributed to the peer-led HIVST intervention without a comparison group. Future studies, preferably randomized controlled trials, are warranted to tease out the relative effect of this intervention on HIVST uptake and linkage to HIV care in fishing community settings, prior to eventual roll-out and scale-up of this promising intervention. Our study could also have benefitted from qualitative interviews to explore the feasibility and acceptability of the intervention, and this presents a limitation, since we only relied on quantitative measures as reported in this paper.

The other limitation is that this study relied on the goodwill of trained peer-leaders to distribute HIV self-test kits to men who had been enrolled into the study. We did not have control over when and where the peer-leaders gave out the kits to their social network members or how much information they gave them regarding the HIVST processes. If peer-leaders gave limited information to their social network members, this could have affected men’s ability to understand the need for confirmatory HIV testing or to link into HIV soon after their HIV-positive diagnosis. This could explain why only 52% of the men interviewed at follow-up indicated that they received information from their peer-leaders about the existing referral networks in the case of a HIV-positive self-test result. These findings suggest a need for developing an intervention checklist (with key intervention talking points) that peer-leaders can use to ensure that they have passed on all the information, as needed, to their members.

Also, while we aimed to enroll only HIV-negative men and men with unknown HIV status, we ended up with 35 men who were already HIV-positive, and who, by implication, should not have been enrolled into the study. This resulted from our reliance on self-reports of HIV-status during the time of screening for participants’ eligibility. However, at the follow-up visit, when we got to know that there were HIV-positive men who had been erroneously enrolled into the study, we conducted post-intervention qualitative interviews with them to understand why they lied that they were HIV-negative when they knew that they were already living with HIV, some of whom already enrolled in HIV care. Some of the men reported that they were not told by their peer-leaders that we were strictly enrolling men with HIV-negative or unknown HIV status while others reported that they doubted their initial HIV-positive diagnoses and wanted to use the self-test kits to “confirm” their original HIV-positive status. The latter assertion calls for a need to educate potential users about the role of HIV self-tests as HIV diagnostic tests that should only be used by those who are either HIV-negative or do not know their HIV status, followed by confirmation of any reactive HIVST results via the national HIV testing algorithm. Detailed results from the post-intervention qualitative assessment will be reported in a follow-on paper.

It is important to note that our findings about confirmatory HIV testing and linkage to HIV care were not only based on self-reports (we did not verify this information at the health facilities) but also on small numbers of men who were newly diagnosed with HIV. Given that there were only sixteen men who were newly diagnosed with HIV, we were unable to compute any inferential statistics since these would largely be unreliable. Thus, our findings on the preliminary effects of the intervention should be interpreted with caution. As noted earlier, large follow-on studies are warranted to address the statistical limitations posed by small numbers but also to address the other limitations posed by using a single-arm, before-after study design as described in this paper. Besides, the use of quota sampling procedures in recruiting men from the existing social networks posed a limitation with regard to the generalizability of study findings. In general, because respondents were selected through referral from a peer-leader (who had discretion over who to refer or not to refer to the study), it is unlikely that the study findings would apply to the entire groups of men from which the respondents were selected or to all men in the fishing communities. However, we believe that given that men in the fishing communities tend to belong to social/occupational groups, it is likely that our findings reflect the HIV testing behaviors of other men in the fishing communities.

Finally, we did not collect data on the socio-demographic characteristics of the peer-leaders or any process data about the peer-leaders’ distribution of HIV self-test kits to their social network members (beyond what is already captured in this paper). Thus, we are unable to tell if there was any variability in the distribution of the kits that could probably be linked to their characteristics. However, given that the peer-leaders distributed 98% of the kits that were given to them by the study team, it is unlikely that there would have been any observed variability in the way the peer-leaders distributed the kits. Nevertheless, further research is warranted to understand the intervention implementation processes and characteristics of the peer-leaders to inform the design and implementation of similar interventions in the future. Despite these limitations, we believe that this study provides additional evidence on the role of peer-led HIVST in improving HIV testing behaviors among men in hard-to-reach fishing communities. This study is one among a few peer-led HIVST studies that have been conducted in Ugandan fishing communities and can thus help to contribute to the design and implementation of future social network-based, HIVST interventions that are suitable to the fisherfolk mode of life.

## Conclusions

Our peer-led HIV self-testing intervention was feasible and acceptable and identified nearly one-third of previously undiagnosed HIV-positive men, seven in ten of whom were eventually linked to HIV care. However, given the small sample size and lack of a comparison group, we recommend that additional research, preferably including a randomized controlled trial, and spanning fishing communities in diverse settings, be conducted prior to the adoption of this promising intervention.

## Data Availability

The datasets used and/or analyzed during the current study are available from the corresponding author on reasonable request.
